# Human Papillomavirus (HPV) Vaccination: Progress, Challenges, and Future Directions in Global Immunization Strategies

**DOI:** 10.3390/vaccines12111293

**Published:** 2024-11-19

**Authors:** Francesco Branda, Grazia Pavia, Alessandra Ciccozzi, Angela Quirino, Nadia Marascio, Simona Gigliotti, Giovanni Matera, Chiara Romano, Chiara Locci, Ilenia Azzena, Noemi Pascale, Daria Sanna, Marco Casu, Giancarlo Ceccarelli, Massimo Ciccozzi, Fabio Scarpa

**Affiliations:** 1 Unit of Medical Statistics and Molecular Epidemiology, Università Campus Bio-Medico di Roma, 00128 Rome, Italy; chiara.romano@unicampus.it (C.R.); m.ciccozzi@unicampus.it (M.C.); 2 Unit of Clinical Microbiology, Department of Health Sciences, “Magna Græcia” University of Catanzaro-“Renato Dulbecco” Teaching Hospital, 88100 Catanzaro, Italy; graziapavia@unicz.it (G.P.); quirino@unicz.it (A.Q.); nmarascio@unicz.it (N.M.); s.gigliotti@unicz.it (S.G.); mmatera@unicz.it (G.M.); 3 Department of Biomedical Sciences, University of Sassari, 07100 Sassari, Italy; aciccozzi@uniss.it (A.C.); c.locci3@phd.uniss.it (C.L.); darsanna@uniss.it (D.S.); 4 Department of Veterinary Medicine, University of Sassari, 07100 Sassari, Italy; iazzena@uniss.it (I.A.); npascale@uniss.it (N.P.); marcasu@uniss.it (M.C.); 5 Department of Chemical Physical Mathematical and Natural Sciences, University of Sassari, 07100 Sassari, Italy; 6 Department of Public Health and Infectious Diseases, University Hospital Policlinico Umberto I, Sapienza University of Rome, 00161 Rome, Italy; giancarlo.ceccarelli@uniroma1.it

**Keywords:** human papillomavirus (HPV), HPV vaccination, cancer prevention, global immunization strategies, vaccine hesitancy, public health policy, next-generation vaccines, personalized medicine in vaccination

## Abstract

Human papillomavirus (HPV) is a widespread viral pathogen, responsible for a significant burden of cervical and other cancers worldwide. Over the past decades, the development and widespread adoption of prophylactic HPV vaccines have dramatically reduced the incidence of HPV-related diseases. However, despite the efficacy of these vaccines, global immunization efforts still face several obstacles, including low vaccination coverage in low- and middle-income countries, vaccine hesitancy, and disparities in access to healthcare. This review aims to provide a comprehensive overview of the current state of HPV vaccines, including their mechanisms of action, safety profiles, and real-world efficacy. We will explore the impact of HPV vaccines on cancer prevention, examine the challenges related to vaccine distribution and uptake, and assess the role of public health policies in improving global vaccination rates. Additionally, the review will highlight the latest advancements in therapeutic HPV vaccines, ongoing research into next-generation vaccines, and the potential of HPV vaccination strategies in the context of personalized medicine. By examining these factors, we aim to provide insights into the future directions of HPV vaccination and its role in global public health.

## 1. Introduction

Over the past two decades, the development of prophylactic human papillomavirus (HPV) vaccines represented one of the most significant advancements in cancer prevention [1]. While cytology-based screening dominated cancer prevention efforts in the 20th century, the current and future landscape of HPV-related cancer prevention is shifting towards a greater reliance on vaccination and molecular screening tests [2,3]. HPV, a highly prevalent sexually transmitted pathogen, is a major contributor to the global burden of cervical and other HPV-related cancers [4,5]. Persistent infection with high-risk oncogenic HPV (HR-HPV) types 16 and 18 is responsible for approximately 70% of cervical cancer cases worldwide [6]. HPV is also implicated in other anogenital cancers, as well as a rising number of oropharyngeal cancers [7]. Despite significant advances, cervical cancer remains a leading cause of cancer-related deaths among women, particularly in low- and middle-income countries (LMICs), where access to screening and treatment is often limited [4]. Since the introduction of the quadrivalent and bivalent HPV vaccines in 2006, clinical trials and post-licensure studies have shown these vaccines to be highly effective in preventing infections with high-risk HPV types, as well as reducing associated precancerous lesions and invasive cancers [1,8]. The World Health Organization (WHO) recognized HPV vaccination as a key component in its global strategy to eliminate cervical cancer [1]. While prophylactic vaccines have been successful in preventing HPV infection, they are most effective when administered before viral exposure, typically to preadolescent populations. Consequently, they do not provide therapeutic benefits to those already infected with HPV or those who have developed HPV-related lesions or cancers [9]. However, these vaccines can help prevent new infections with different HPV types or re-infections with the same type [9]. This limitation has spurred growing interest in the development of therapeutic HPV vaccines, which aim to treat existing HPV infections and related malignancies [10]. Recent findings from a systematic review and meta-analysis conducted by the International Agency for Research on Cancer (IARC) revealed that only 54% of women with advanced cervical intraepithelial neoplasia (CIN) grades 2 and 3 who received therapeutic HPV vaccines experienced lesion regression [10]. In response, the WHO is developing product performance characteristics to enhance the efficacy of therapeutic HPV vaccines [11]. Despite impressive advancements, global vaccination efforts continue to face significant obstacles. Vaccine distribution and uptake are particularly limited in LMICs, where healthcare infrastructure is scarce, and vaccination coverage remains low [12,13]. These challenges are further complicated by vaccine hesitancy, misinformation, and socioeconomic and gender inequities, highlighting the need for targeted interventions to improve access and ensure equitable healthcare delivery [12,13].

This review aims to provide a comprehensive analysis of the current landscape of HPV vaccination, with a focus on both prophylactic and therapeutic vaccines, including their mechanisms of action, safety profiles, and real-world effectiveness. Additionally, it will explore the challenges impeding global vaccination efforts, the role of public health policies in enhancing vaccination rates, and the potential of vaccines and personalized strategies in the future of HPV immunization. By addressing these factors, this review seeks to offer insights into the evolving role of HPV vaccination in cancer prevention and global public health.

## 2. HPV Infection: General Aspects and Virology of HPV

HPV belongs to the *Papillomaviridae* family and is a small, non-enveloped virus with a diameter of approximately 55 nm [14]. The viral genome consists of a single, circular, double-stranded DNA molecule. Its genome is divided into three functional regions. The first region, the non-coding upstream regulatory region (URR), also called the long control region (LCR), represents the most variable portion of the viral genome. This regulatory region contains the p97 core promoter and enhancer and silencer elements, which play pivotal roles in modulating the transcriptional activity of open reading frames (ORFs) and orchestrating the replication of viral DNA. The second region, encoding the early (E) proteins, comprises ORFs E1, E2, E4, E5, E6, and E7. These genes are essential for viral replication, persistence, and the oncogenic transformation of infected cells, primarily through their interference with cellular regulatory mechanisms [15]. Lastly, the late region (L) encodes the major structural proteins L1 and L2, which assemble to form the viral capsid, facilitating the encapsulation and protection of viral DNA [14]. Its capsid comprises 72 capsomers, predominantly formed by the major structural protein L1, which oligomerizes into pentameric subunits [16]. Additionally, the capsid incorporates approximately 12 copies of the minor structural protein L2, which plays a role in viral assembly and infection [17].

HPV types are classified based on their genetic sequences, with new or unknown types identified when the E6, E7, and L1 ORF sequences show less than 90% homology to known types [18]. This genetic organization underlies the virus’s ability to replicate and its involvement in oncogenesis, making HPV a key target for vaccine development and cancer prevention efforts.

Phylogenetically, HPVs are categorized into five main genera: α, β, γ, δ, and µ, based on a minimum of 10% variation in the nucleotide sequence of their ORF L1. The α genus primarily infects the genital and oropharyngeal mucosal cells and includes the oncogenic HPV types linked to malignant cervical carcinoma [19,20]. Variants of HPV, which are defined by less than 3% variation in their L1 sequences, often exhibit tissue specificity; certain variants are associated with premalignant or cancerous lesions, while others are linked to benign conditions. Clinically, HPVs are classified according to their oncogenic potential as either “low-risk” (LR-HPV) or “high-risk” (HR-HPV) types [21]. Approximately 90% of invasive cervical cancers are of the squamous cell type, with less than 5% being adenocarcinomas, both of which are associated with distinct HPV types. Rare histological subtypes account for the remaining cervical carcinoma [19]. HPV types 16 and 18 are the most clinically significant in causing genital malignancies, particularly cervical cancer. HPV6, initially identified in benign genital warts (condyloma acuminata), along with HPV11, is categorized as a low-risk type and is commonly associated with non-malignant lesions such as laryngeal papillomas and genital warts [22]. In contrast, HPV16 and 18 are recognized as HR-HPV types, frequently detected in high-grade cervical dysplastic lesions as well as most invasive cervical cancers. These HR-HPV types can facilitate the progression from high-grade precancerous lesions to invasive carcinoma [7,21,22]. HPV16 is recognized as the most oncogenic human papillomavirus type, contributing to cervical and other anogenital cancers, as well as oropharyngeal cancers. This high-risk genotype is further divided into four major phylogenetic lineages—A, B, C, and D—each with distinct biological and epidemiological characteristics. These lineages are classified into nine variant sublineages: A1, A2, A3, A4, B1, B2, C, D1, and D2. Such genetic diversification has implications for pathogenesis and global distribution patterns, influencing viral persistence, immune evasion, and oncogenic potential across populations. A landmark study by Mirabello et al. [23], which analyzed HPV16 variants across different geographical and ethnic groups, provided compelling evidence of HPV16’s oncogenic dominance. This research included an extensive cohort of over 3200 women with confirmed cervical cancer and demonstrated that specific HPV16 lineages, especially those within the A lineage, were significantly associated with increased cancer risk and were predominant in various global populations studies, which have shown that lineage A variants tend to exhibit higher oncogenicity and are more commonly linked to severe dysplasia and carcinoma in situ than other lineages, suggesting a potential influence of viral genetics on disease progression [23]. Other HR-HPV types, such as 31, 33, 45, 52, and 58, also exhibit oncogenic potential, although they are less frequently implicated in cervical cancer compared to HPV16 and 18. Together, these two types are the most prevalent globally, found in both symptomatic and asymptomatic women, and are associated with the majority of invasive cervical cancer cases worldwide [7,22,24,25].

## 3. Global and Local Burden of HPV Infection: Epidemiology, Prevention, and Socioeconomic Impact

HPV infection remains a significant public health challenge worldwide, with substantial regional variations in both prevalence and impact. These differences reflect disparities in vaccination coverage, public health interventions, and socioeconomic conditions. Globally, the burden of HPV infection varies significantly across regions, reflecting differences in vaccination coverage, public health interventions, and socioeconomic conditions. More than 200 HPV types have been identified, of which about 14 are classified as high-risk because of their association with cancers [7]. According to the meta-analysis by Clifford et al. [26], vaccinating against HPV16 and 18 should prevent over 70% of worldwide invasive cervical cancer (ICC). Cervical cancer is the fourth most common cancer among women, with around 660,000 new cases reported in 2022 and approximately 350,000 deaths occurring in the same year. Notably, 94% of these deaths occurred in low- and middle-income countries, highlighting disparities in access to vaccination, screening, and treatment services. The highest rates of cervical cancer incidence and mortality are found in sub-Saharan Africa, Central America, and South-East Asia, areas where the economic burden of HPV-related diseases is exacerbated by limited resources and healthcare infrastructure. Women living with HIV are particularly vulnerable, being six times more likely to develop cervical cancer compared to the general population, and around 5% of all cervical cancer cases are directly attributable to HIV [27]. These disparities underline the critical need for effective prevention strategies, including HPV vaccination and regular screening, to mitigate the public health impact of cervical cancer in the most affected regions.

The introduction of HPV vaccination programs in high-income countries has helped reduce the prevalence of high-risk HPV types. A systematic review by Drolet et al. [28] demonstrated a significant reduction in HPV types 16 and 18 among vaccinated populations, particularly in regions with high vaccine prevalence. However, challenges remain in LMICs, where limited access to vaccines continues to hinder progress. These disparities in vaccine coverage result in a persistently high burden of HPV-related diseases in regions with limited health resources, highlighting the need for comprehensive efforts to improve vaccine access and coverage. 

In the Italian context, progress has been made in HPV vaccination, particularly among adolescents, through national vaccination campaigns targeting 12-year-old girls. Data from the Italian Ministry of Health indicate that vaccination coverage for this group reached about 70% by 2020 [29]. Nevertheless, regional disparities in vaccine uptake persist, with Southern Italy reporting lower coverage compared to Northern regions. These differences are often associated with socioeconomic factors, healthcare accessibility, and varying levels of awareness regarding the risks of HPV and the benefits of vaccination. The prevalence of HPV in Italy aligns with global patterns, with HPV types 16 and 18 being the most frequently detected high-risk types in cervical intraepithelial neoplasia (CIN) and cervical cancer cases. Carozzi et al. [30] noted the predominance of these types in Italian populations, emphasizing the ongoing need for targeted vaccination efforts.

Organized screening programs have played a crucial role in reducing the incidence of cervical cancer in Italy, particularly through the use of HPV-based screening methods. Ronco et al. [31] highlighted the effectiveness of these programs in detecting high-risk infections early, thus preventing progression to invasive cancers. The shift from cytology-based screening to HPV testing has proven to be a valuable public health strategy that can enhance early detection and improve outcomes for women at risk. In addition to cervical cancer, HPV is also associated with other anogenital and oropharyngeal cancers, particularly among men. Recent studies have reported an increase in the incidence of HPV-related oropharyngeal cancer in Western countries, suggesting the need to expand vaccination efforts to include males [32]. This changing epidemiological landscape has led to renewed calls for gender-neutral vaccination policies and public health campaigns targeting both men and women, with the goal of reducing overall HPV transmission. Addressing the broader implications of HPV-related cancers is critical, as the burden of disease extends beyond individual health outcomes to impact health systems and economies. The economic burden of HPV-related diseases is particularly significant in regions with limited resources for vaccination and screening programs. Studies such as that conducted by Llave et al. [33] have shown that HPV vaccination, when combined with regular screening, is a cost-effective strategy to reduce the long-term healthcare costs associated with HPV-related cancers. Investing in preventive measures, such as vaccines and screening programs, offers a sustainable pathway to mitigate the public health impact of HPV by reducing morbidity and mortality rates over time.

As we approach 2030, the year in which the WHO has set out to achieve key targets for the elimination of cervical cancer as a public health problem, there are significant challenges as well as opportunities. Achieving the goals of 90% vaccination coverage, 70% of women screened, and 90% of women treated will require a concerted effort by governments, international organizations, and civil society. The increasing availability of safe and effective HPV vaccines represents a great opportunity, but barriers to access must be addressed, especially in LMICs. The COVID-19 pandemic has strained global health systems, and this has further slowed the implementation of vaccination and screening programs. However, accelerating these post-pandemic programs is critical to ensure that the gains made so far are not lost and to prevent millions of lives from continuing to be at risk.

## 4. Diagnostic Approaches and HPV Prevention: Microbiological Testing and Vaccination Strategies

Several techniques have been reported for the identification of HPV. One of the older methods is based on the ability of HPV DNA to integrate into human DNA. The FISH method relies on staining portions of the viral episomal genome. Oral keratinocytes or cells from cervical cancer have been reported to be positive for the HPV genome by the FISH technique [34]. Among female patients with very early cervical dysplasia, the evaluation of HPV types 16 and 18 by quantitative RT-PCR (qRT-PCR) has been demonstrated as a very useful and sensitive technique [35]. Regarding epigenetic markers of HPV-associated diseases, it has been demonstrated that methylation of CpG sites within the genome is associated with both early and late phases of carcinogenesis. Usually, hypermethylated regions are thought to be associated with healthy or early dysplastic lesions, while hypomethylation might be found within advanced cancer phases [36]. For several years, viral oncogenes such as E6 and E7 have been assessed in cervical swabs using NASBA and transcription-mediated amplification techniques as biomarkers for cervical pre-cancerous lesions [37]. The identification of HPV E6/E7 mRNA was further improved by the association of FISH and flow cytometry techniques. Such an association of methods showed high sensitivity and specificity among women with high-grade intraepithelial lesions [38]. In dealing with protein biomarkers of HPV, a pivotal role has been played by p16 ^ink14^ and p16 ^ink4a^, which can be tested by ELISA and immunohistochemical techniques [39]. Very recently, novel diagnostic approaches emerged to exploit nucleic acid amplification-based biosensors for their advantages of high sensitivity, rapid operation, and portability, all features very useful for quick testing [40]. Third-generation sequencing has enabled the detection of multiple clonal integration events and the identification of critical viral integration sites within the cancer genome. The extended read lengths offered by these technologies represent a significant advancement, allowing for real-time sequencing with unparalleled resolution. These capabilities mark a substantial improvement in unraveling the complex interactions between viral oncogenes, viruses, and cancer [41]. Table 1 summarizes the molecular assays used for diagnosis of HPV infection.

The tangled relationship between HPV infection immunity and cancer development made the discovery, the trial phase, and clinical use introduction of HPV vaccines difficult. Two types of vaccines were historically linked to HPV: the first aiming at infection prevention and the second associated with control of viral progression and pre-cancer and cancer evolution [46]. The use of prophylactic vaccines started in 2006, aiming to immunize adolescents. A quadrivalent vaccine targeting the two major high-risk variants, HPV-16 and -18, and the low-risk genotypes HPV-6 and -11, was introduced following US Food and Drug Administration approval in June 2006 for use in women 9–26 years of age [47]. After, a second-generation vaccine introduced in 2014 was able to target HPV-6, -11, -16, and -18, as well as HPV-31, -33, -45, -52, and -58 [48]. Such novel vaccines broadened the involved population, including both adolescent girls and boys (https://www.merckvaccines.com/gardasil9, accessed on 30 October 2024). Gardasil 9 PI exhibited a significant prophylactic effect in the population with large vaccine coverage, while lower-income countries were not able to exploit the advantages of such a vaccine due to low coverage [49]. On the contrary, oropharyngeal neoplastic lesions were not so well controlled in comparison with cervical cancer and the prevalence of such an illness was continuing to increase regardless of the use of a nonavalent vaccine [50]. Prophylactic HPV vaccines including Gardasil^®^ and Cervarix^®^ are virus-like particles (VLPs) that can stimulate antibody responses against L1 protein. Such antibodies inhibit virus entry into the epithelial cells; thus, the virus cannot cause an infection. Unfortunately, these vaccines have very modest effects against established infections [50]. Recently, therapeutic vaccines designed to target HPV-infected cells by inducing cell-mediated immune responses had significant potential as effective strategies for treating CIN lesions and achieving viral clearance [51]. A wide range of therapeutic HPV vaccines has been investigated, including protein-based vaccines, viral-vectored vaccines, bacterial-vectored vaccines, as well as DNA-based and cell-based vaccines [50]. While none of these vaccines has yet received regulatory approval for clinical application, several have advanced to phase II or III clinical trials [52]. Also, the E2, E5, E6 and E7 proteins are potential targets of therapeutic HPV vaccines, due to their relevant role in oncogenesis. Therapeutics vaccines, such as AD26 and AD35, are expressing both early and late proteins (E2, E6, E7) of HPV16/18, and it appears that they stimulate a strong CD8 T cell specific response without CD4 upregulation. In an animal model, the administration of fusion proteins of E2 with the N-terminal portion of E6 and E7 produced high immunogenicity. Such a vaccine would be useful for every phase of disease, from the early stages to the late neoplastic evolution of HPV infection. Several studies evaluating therapeutic vaccines included subjects with CIN2 and CIN3, and lesion regression was the vaccine efficacy endpoint. Two studies focused on patients with advanced cervical cancer. In these latest studies, the vaccine efficacy endpoints were considered the clinical response or patient survival. In the investigations with CIN2 and CIN3 lesions, therapeutic vaccines were associated with moderate but significant lesion improvements. On the contrary, in patients with advanced cervical cancer, therapeutic vaccines did not produce significant beneficial effects [52].

## 5. Genetic Aspects of HPV Infection: Viral Variants and Individual Predisposition

Human papillomavirus (HPV) infection is shaped by a complex interplay between viral characteristics and host genetic predispositions, which together influence the likelihood of viral persistence, progression to malignancy, and clinical outcomes [53]. HPV is a highly diverse virus with over 200 genotypes, divided into low-risk and high-risk categories, with high-risk types—particularly HPV-16 and HPV-18—linked to most HPV-related cancers, including cervical, anal, and oropharyngeal cancers [54]. However, despite the prevalence of these high-risk infections, not all individuals develop cancer, suggesting that host genetic factors significantly affect disease susceptibility and progression [55].

HPV genetic diversity plays a key role in infection outcomes by influencing the virus’s ability to evade immune responses and promote oncogenesis. High-risk HPV types like HPV-16 show significant oncogenic potential due to viral oncoproteins such as E6 and E7, which inhibit critical tumor suppressor proteins like p53 and retinoblastoma (Rb) [56,57]. Within high-risk types, genetic variations among viral isolates exist. Some intratype variants, or “lineages”, such as specific ones of HPV-16, are more common in invasive cancers than others, highlighting how genetic diversity contributes to differing clinical outcomes [58,59]. Certain viral variants are more likely to integrate into the host genome, a critical step in the malignant transformation process that can disrupt normal cellular regulation and accelerate oncogenesis. Understanding HPV genetic diversity is essential for refining diagnostic approaches and tailoring preventive strategies, such as next-generation vaccines targeting the most oncogenic variants [60,61].

Host genetic variability also plays a crucial role in HPV infection outcomes. A primary factor is genetic variability in the immune system, particularly in genes involved in antigen presentation, immune surveillance, and viral clearance. The human leukocyte antigen (HLA) system is essential for recognizing viral antigens and presenting them to T cells [56,62]. Certain HLA class I and II alleles are associated with increased susceptibility to HPV persistence or enhanced viral clearance; for example, individuals with specific HLA class II haplotypes, such as HLA-DRB113 and HLA-DQB106, have a higher likelihood of clearing infections, while others, such as HLA-DRB1*15, are associated with increased risk of persistent infection and progression to cervical cancer [63,64]. Beyond the HLA system, genetic polymorphisms in other immune-related genes affect HPV infection outcomes. Variants in genes encoding cytokines, chemokines, and their receptors, such as interleukin-10 (IL-10) and tumor necrosis factor-alpha (TNF-α), modulate the inflammatory response to HPV [65]. For instance, individuals with polymorphisms leading to increased expression of IL-10, an anti-inflammatory cytokine, may experience a dampened immune response, favoring infection persistence. Conversely, TNF-α polymorphisms associated with a more robust inflammatory response may enhance viral clearance but also contribute to tissue damage and inflammation that could promote carcinogenesis [66].

HPV’s ability to cause cancerous transformation is closely linked to its interference with the host’s DNA repair mechanisms and cell cycle regulation. Host genetic polymorphisms in genes involved in these processes significantly affect the outcome of HPV infection. The integration of viral DNA into the host genome can provoke genomic instability and mutation accumulation [67,68]. Host genes involved in DNA repair, such as those in the nucleotide excision repair (NER) pathway, are critical in correcting these errors. Variants in DNA repair genes, such as XRCC1 and ERCC1, have been associated with an increased risk of HPV persistence and cancer progression. Individuals with reduced DNA repair capacity may be more vulnerable to HPV-induced genomic instability, raising their likelihood of malignant transformation [69,70]. Host polymorphisms in cell cycle control genes like TP53 further modulate susceptibility to HPV-related cancers [71]. Hormonal factors also influence HPV-related cancer risk, especially in hormone-sensitive tissues like the cervix. Estrogen, for instance, can stimulate the proliferation of HPV-infected epithelial cells, increasing the chances of malignant transformation. Genetic variants in estrogen receptor genes and those involved in estrogen metabolism, such as CYP1A1 and CYP1B1, have been associated with an increased risk of cervical cancer in HPV-infected women [72,73]. Understanding these genetic aspects of HPV infection offers the potential to revolutionize approaches to prevention, diagnosis, and treatment. Genetic screening could help identify individuals at higher risk of persistent infection or cancer progression, allowing for earlier and more tailored interventions. For instance, individuals with certain HLA haplotypes or deficiencies in DNA repair mechanisms may benefit from more frequent screening, personalized vaccination strategies, or targeted therapies [74] designed to mitigate their specific risks (https://www.news-medical.net/news/20240412/New-genetic-variants-could-raise-a-womans-risk-of-cervical-cancer-from-HPV-infections.aspx, accessed on 30 October 2024).

In conclusion, the genetic aspects of HPV infection encompass a wide range of factors, including viral genetic diversity, host immune response, DNA repair capabilities, and hormonal regulation. The interaction between these factors ultimately determines the outcome of HPV infection, influencing whether the virus is cleared, persists, or progresses to cancer. By continuing to explore these genetic influences, researchers can develop more effective, personalized strategies to combat HPV and reduce the global burden of HPV-related cancers.

## 6. Clinical Manifestations of HPV Infection

**Figure 1:**
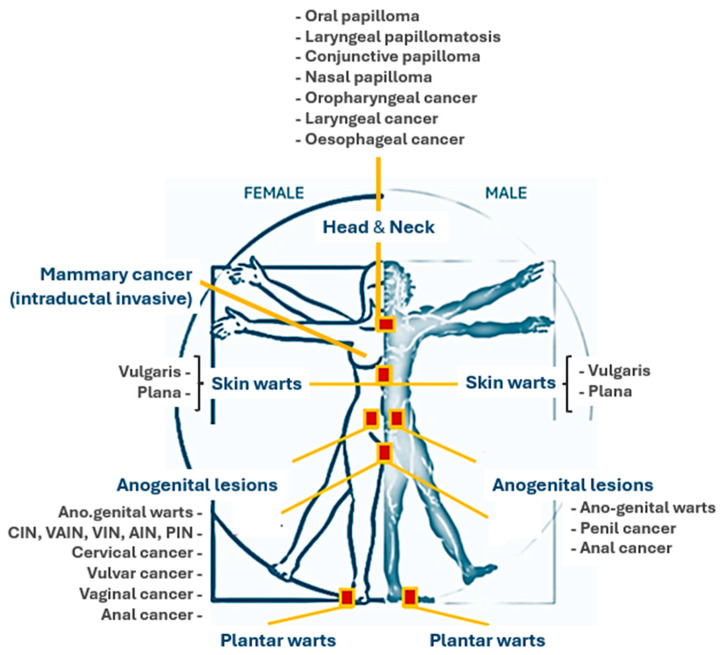
Anatomical sites of main HPV-related lesions.

Human papillomavirus infections present with varying clinical manifestations depending on the anatomical site affected (Figure 1), largely due to the site-specific tropism of certain viral genotypes (Table 2).

While the prognosis for HPV infection is generally good, recurrences are frequent. Wart treatments, though numerous, are not consistently effective, often necessitating repeated applications. HPV infection can also cause vulvar and cervical intraepithelial neoplasia and cervical cancer. Some women remain at elevated risk for vaginal and anal cancers, with immunocompromised individuals facing the highest risk of malignancy [79]. A diagnosis of HPV carries a 5–20% risk of co-infection with other STIs like gonorrhea or chlamydia.

## 7. Conclusions and Future Directions

HPV prevention, primarily through vaccination, represents one of the most impactful advancements in global public health, significantly reducing the burden of HPV-associated diseases such as cervical cancer. Nevertheless, several key challenges continue to hinder the widespread success of HPV immunization programs, especially in LMICs, where cervical cancer incidence and mortality remain high. Addressing the gaps in vaccine coverage, overcoming vaccine hesitancy, and ensuring equitable access to healthcare and vaccination services are crucial steps for improving global HPV prevention outcomes.

Moving forward, enhancing HPV vaccination coverage worldwide will require multifaceted strategies that encompass not only healthcare access improvements but also community engagement and education. Public health initiatives should focus on overcoming cultural and logistical barriers to vaccine acceptance, particularly in communities with historically low immunization rates. Moreover, integrating HPV vaccines into routine immunization schedules and expanding access to low-cost or subsidized vaccine options in LMICs could play a pivotal role in closing the immunization gap.

From a research point of view, there is significant promise in the development of next-generation HPV vaccines that could potentially cover a broader range of HPV types or offer extended immunity with fewer doses [80]. Research into therapeutic HPV vaccines is also advancing, with potential applications for individuals already infected or with HPV-related lesions, which could further reduce the long-term burden of HPV-associated diseases. Additionally, exploring genetic factors linked to individual susceptibility to HPV infection and progression may contribute to more personalized HPV prevention approaches in the future (https://www.news-medical.net/news/20240412/New-genetic-variants-could-raise-a-womans-risk-of-cervical-cancer-from-HPV-infections.aspx#:~:text=We%20found%20certain%20genetic%20variants,risk%20of%20developing%20cervical%20cancer, accessed on 30 October 2024). The convergence of HPV vaccination strategies with advances in personalized medicine, such as predictive models for HPV susceptibility and customized prevention plans, offers new potential for highly targeted and effective HPV prevention strategies. Longitudinal studies assessing the impact of HPV vaccines on cancer prevention and real-world vaccine efficacy are essential to guiding these innovations.

In conclusion, while significant strides have been made in the fight against HPV, a coordinated global response that emphasizes accessibility, education, and continued research into vaccine improvements is necessary. Strengthening collaboration between governments, healthcare providers, and international organizations will be essential to maximizing the global health benefits of HPV vaccination. As we advance, HPV prevention strategies hold the potential to not only reduce the incidence of HPV-related cancers worldwide but also to serve as a model for preventing other infectious diseases with significant public health impacts.

## Figures and Tables

**Table 1:** Molecular assays for diagnosis of HPV infection.

Type of Assays	Tests	Targets	Sensibility (%)	Specificity (%)	References
**DNA-based assay**	Digene Hybrid Capture 2 high-risk HPV DNA test Cervista HPV HR and Genfind DNA extraction kit Cobas HPV test	13 high-risk HPV types using multigene probes L1, E6, E7 genes and 14 high-risk HPV types L1 gene, 14 high-risk HPV types, and HPV16 and Hpv18	92.86 (CIN2) 89.47 (CIN3) 98.4 82.14 (CIN2) 78.95 (CIN3)	43.67 (CIN2) 42.86 (CIN3) 85.2 66.46 (CIN2) 65.42 (CIN3)	[42] [43] [42]
**HPV genotyping assays**	Cervista HPV16/18	Detects and differentiates HPV16 and HPV18	77%		[44]
**E6/E7 mRNA-based assay**	Aptima HPV assay	E6/E7 viral mRNA and 14 high-risk HPV types	95.5%	94.5%	[45]

**Table 2:** Clinical manifestations of HPV infection.

HPV Type and Disease Association [75]	Characteristics of HPV Related Diseases [76,77,78]	Clinical Presentation [76,77,78]
**Anogenital Warts** **—————-** A total of 90% are caused by HPV types 6 or 11. Other types are 30, 42, 43, 45, 51, 54, 55, and 70.	**Localization**: Primarily affecting moist areas such as the perianal region, vaginal introitus, vagina, labia, and vulva. However, they can also arise on dry skin surfaces, including the penile shaft. **Morphology**: The spectrum ranges from smooth papular warts to keratotic warts, the latter resembling common cutaneous warts due to their thickened, irregular texture. Flat condylomata (squamous intraepithelial neoplasia) typically manifest as white, plaque-like lesions, most commonly on the cervix but also potentially involving the vulva, anus, and male genitalia. **Giant condyloma:** This is a malignant variant classified as verrucous carcinoma. Localization: typically affects the glans penis, perianal region, and foreskin. Morphology: large, cauliflower-like masses, often exhibiting abscess formation, fistula development, and local invasion.	Typically, they occur several months after HPV inoculation. They pursue a slow, indolent course, often spreading through autoinoculation between adjacent skin surfaces. Condylomata acuminata are frequently asymptomatic but can cause pruritus. Bleeding may arise from lesion confluence and irritation by clothing.
**Cervical HPV** **Infection and Disease** **—————-** HPV types **Low-risk** 6, 11, (31, 33, 35, 42, 43, 44, 45, 51, 52, 74) **High-risk** 16, 18, (6, 11, 31, 34, 33, 35, 39, 42, 44, 45, 51, 52, 56, 58, 66)	**Localization:** cervical region of uterus. **Morphology:** low-grade squamous intraepithelial lesion or high-grade squamous intraepithelial lesion (Papanicolaou smear screening). Acetowhite changes and abnormal vascular patterns are indicative of HPV-associated dysplasia (3–5% acetic acid and colposcopy). HSIL can progress to invasive cervical cancer.	Most cervical HPV infections are latent or subclinical and therefore asymptomatic. Symptoms at cancer stage may include intermenstrual or postcoital bleeding, dyspareunia, and pelvic fullness.
**Anal Cancer** **—————-** HPV types 16, 18, (31, 45, 33, 35, 39, 51, 52, 56, 58, 66, 68, 70)	**Localization:** anal region. **Morphology:** HPV-associated inflammation can lead to anal intraepithelial neoplasia (AIN). AIN is graded I-III based on the degree of abnormality in squamous cell differentiation and maturation, mitotic activity, nuclear membrane changes, and the depth of these abnormalities within the epithelium. AIN can progress to invasive squamous cell carcinoma (SCC) in approximately 10–11% of cases. A history of anorectal warts is more common in homosexual men (50%) with anal SCC compared to women and heterosexual men (20%)	Asymptomatic or a range of symptoms including anal bleeding, anal or pelvic pain, weight loss, the sensation of anal or rectal mass, anal irritation, tissue prolapse, flatus or fecal incontinence, and constipation. SCC is often misdiagnosed as hemorrhoids.
**Non-anogenital Mucosal Disease** **—————-** HPV types 6, 11	Involve various non-anogenital mucosal surfaces, including the nares, mouth, larynx, and conjunctiva. Oral warts are indicative of HPV infection of the oral mucosa. Focal epithelial hyperplasia, a disseminated HPV infection of the oral mucosa, is primarily associated with HPV types 13 and 32 and may exhibit a familial predisposition.	Oral warts are relatively common but often subtle and easily missed.
**Non-genital Cutaneous HPV** **—————-** Verruca vulgaris 1, 2, 4, 7 Palmoplantar warts 1, 2, 4, 63 Verruca plana 2, 3, 10	**Verruca Vulgaris:** These warts typically occur on keratinized skin, presumably at the site of viral inoculation. Autoinoculation can lead to the development of adjacent warts. They present as circumscribed, rough, hyperkeratotic papulonodular lesions or plaques with irregular, scaly surfaces, most commonly on the hands, fingers, feet, and knees.	While generally asymptomatic, they can be painful with applied pressure and are typically benign and self-limiting.
	**Palmoplantar Warts:** Affecting the acral surfaces of the hands and feet, these warts are characteristically thick, posing therapeutic challenges. Thrombosed capillaries can appear as small, black “seeds” within the wart.	Deep plantar warts often present as solitary lesions that may become blackened and painful before spontaneously regressing.
	**Verruca Plana:** These frequently manifest as clusters of small plaques (less than 5 mm in diameter) on the face and hands. They typically regress spontaneously within several years.	Often preceded by pruritus or erythema, they can cause significant pigmentary changes.
	**Malignant transformation of skin lesions:** Typically begins in the fourth and fifth decades of life. Premalignant lesions often first appear on sun-exposed areas such as the forehead.	These lesions range in clinical severity from benign papillomas and seborrheic keratoses to premalignant actinic keratoses and squamous cell carcinoma.

## Data Availability

No new data were created in this study.
